# Smooth Muscle Cell MAPK14 Promotes Vascular Calcification During Hyperlipidemic Aging

**DOI:** 10.3390/cells15181625

**Published:** 2026-09-08

**Authors:** Nestor Ishimwe, Yuchi Tu, Chunhui Wang, W. Bart Bryant, Wei Zhang, Yabing Chen, Xiaochun Long

**Affiliations:** 1 Vascular Biology Center, Department of Medicine, Medical College of Georgia, Augusta University, Augusta, GA 30912, USA; nishimwe@augusta.edu (N.I.); drhui666@163.com (C.W.);; 2 Lemole Center for Integrated Lymphatic and Vascular Research, Lewis Katz School of Medicine, Temple University, Philadelphia, PA 19140, USA; 3 Department of Pathology & Laboratory Medicine, School of Medicine, Oregon Health & Science University, Portland, OR 97239, USA; 4 Portland Veterans Affairs Medical Center, Portland, OR 97239, USA

**Keywords:** vascular calcification, vascular aging, MAPK14, RUNX2, vascular smooth muscle cells

## Abstract

Vascular calcification is a hallmark of vascular aging that is accelerated by hyperlipidemia and contributes to adverse cardiovascular outcomes. Although vascular smooth muscle cells (VSMCs) are key mediators of vascular calcification, the signaling pathways linking aging-associated stress to osteogenic remodeling remain incompletely understood. To define the role of MAPK14 (p38α) in vascular aging, we integrated bulk RNA sequencing of young and aged mouse aortas with published single-nucleus RNA sequencing data from VSMC-specific *Mapk14* knockout (KO) mice. Bulk RNA-seq identified age-associated activation of extracellular matrix remodeling, calcification, inflammatory, and senescence-associated gene programs, accompanied by increased p38 MAPK signaling. Single-nucleus RNA-seq further demonstrated that *Mapk14* KO attenuated proliferative, inflammatory, fibrotic, and ossification-associated gene modules in VSMCs. Consistent with these transcriptomic findings, RUNX2 expression was markedly increased in aged aortas, whereas aged *Mapk14* KO mice exhibited reduced RUNX2 expression together with attenuated vascular calcification, fibrosis, and inflammatory cell infiltration compared with wild-type controls. Collectively, these results identify VSMC MAPK14 as an important regulator of vascular calcification during hyperlipidemic aging and demonstrate that MAPK14 deficiency is associated with reduced RUNX2 expression and attenuated fibro-inflammatory vascular remodeling. These findings support MAPK14 as a potential therapeutic target for limiting vascular calcification and associated pathological remodeling during aging.

## 1. Introduction

Vascular aging is characterized by progressive structural and functional remodeling of the arterial wall, including increased stiffness, extracellular matrix (ECM) reorganization, and calcification, which collectively contribute to increased cardiovascular morbidity and mortality [1,2]. Among these features, vascular calcification is a defining hallmark of vascular aging and is now recognized as an actively regulated, cell-driven process rather than a passive degenerative event [3,4]. Vascular smooth muscle cells (VSMCs) play a central role in this process by acquiring an osteogenic phenotype in response to pathological stress. The transcription factor RUNX2 is a master regulator of osteogenic differentiation and is essential for activation of osteogenic gene programs and vascular calcification in VSMCs [5,6]. However, the upstream pathways linking age-associated stress to RUNX2 activation and calcific remodeling remain incompletely defined.

MAPK14, which encodes p38α, a major isoform of the p38 MAPK family, is a stress-activated serine/threonine kinase that integrates diverse cellular stress signals and regulates inflammation, senescence, ECM remodeling, and cell fate decisions. Our prior studies demonstrate that MAPK14 promotes VSMC phenotypic modulation and contributes to neointimal hyperplasia and aneurysm formation, in part through regulation of transcriptional programs involving MRTFA and MYOCD [7,8,9]. In addition, pharmacological inhibition of MAPK14 signaling attenuates vascular calcification in experimental models of chronic kidney disease (CKD), suggesting that MAPK14 may contribute to osteogenic vascular remodeling [10]. Nevertheless, whether MAPK14 regulates RUNX2 expression and promotes vascular calcification during hyperlipidemic aging remains unknown.

Here, we investigated the role of smooth muscle cell MAPK14 in age-associated vascular calcification using aged *Apoe*^−/−^ mice with or without smooth muscle cell-specific MAPK14 deficiency. By integrating bulk and single-nucleus transcriptomic analyses with biochemical and histological characterization, we demonstrate that VSMC MAPK14 promotes vascular calcification accompanied by fibro-inflammatory remodeling during hyperlipidemic aging. MAPK14 deficiency also reduces RUNX2 expression, consistent with suppression of osteogenic signaling. These findings identify MAPK14 as an important regulator of vascular calcification and suggest that targeting MAPK14 may represent a promising therapeutic strategy for limiting vascular calcification and other pathological features of vascular aging.

## 2. Materials and Methods

### 2.1. Experimental Animals

All animal studies were performed in accordance with protocols approved by the Institutional Animal Care and Use Committee at Augusta University. Mice were housed in a temperature-controlled room under a 12 h light/dark cycle and maintained on a normal chow diet. Both male and female mice were used for experimental validation studies. Smooth muscle cell–specific *Mapk14* knockout (KO) mice were generated by crossing *Mapk14*^f/f^ mice with SM22-Cre transgenic mice on an *Apoe*^−/−^ background, as previously described [8]. SM22-Cre; *Apoe*^−/−^ mice were used as wild-type (WT) controls, and SM22-Cre; *Mapk14*^f/f^; *Apoe*^−/−^ mice were used as VSMC–specific *Mapk14* KO mice. Male mice were euthanized at 26, 60, 65, and 86 weeks of age, and female mice were euthanized at 55 and 86 weeks of age. Aortas were then collected for Western blot and histopathological analyses.

### 2.2. Omics Data Analysis

**Figure 1:**
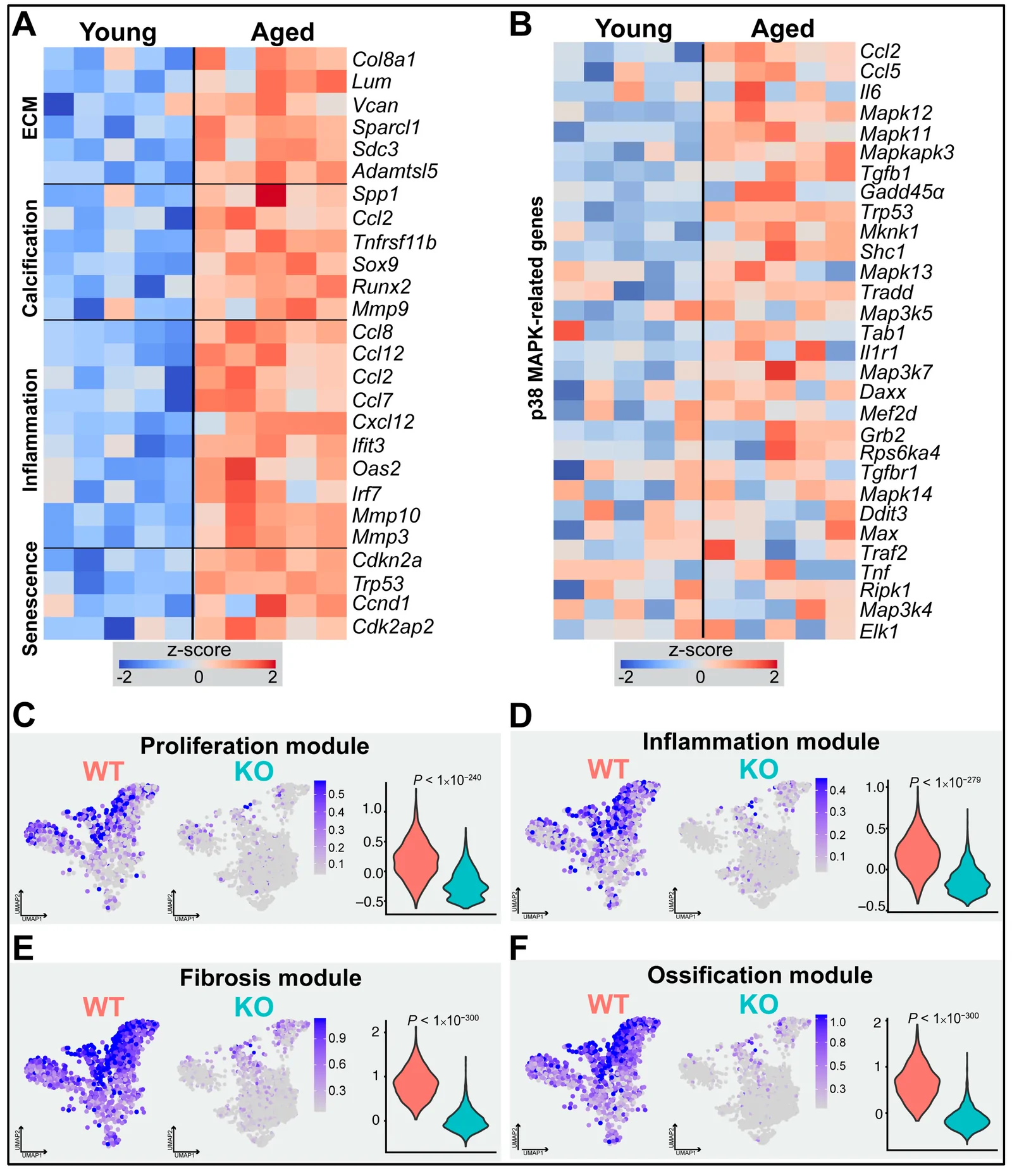
Aging activates MAPK14-associated inflammatory and calcific gene programs in the aorta. (**A**) Bulk RNA-seq analysis showing significant enrichment of extracellular matrix (ECM), calcification, inflammatory, and senescence-associated pathways in aged compared with young mouse aortas. The heatmap shows increased expression of representative genes selected from each enriched pathway (*n* = 5/group). (**B**) Heatmap showing increased expression of genes associated with p38 MAPK signaling in aged aortas compared with young controls (*n* = 5). (**C**–**F**) Single-nucleus RNA-seq analysis of aortas from wild-type (WT) and vascular smooth muscle cell (VSMC)-specific *Mapk14* knockout (KO) mice. UMAP and violin plots demonstrate downregulated proliferation (**C**), inflammatory (**D**), fibrotic (**E**), and ossification (**F**) gene modules in VSMCs from KO mice compared with WT controls.

Bulk RNA-seq and single-nucleus (sn)RNA-seq analyses of mouse aortas were performed using our previously published datasets. Bulk RNA-seq data were obtained from the Gene Expression Omnibus (GSE145972), and snRNA-seq data were obtained from the NCBI Sequence Read Archive (SRA; PRJNA1332774) [9,11]. p38 MAPK signaling-related genes were curated from the BioCarta and Harmonizome databases. The exact gene sets used for module-score analyses are listed in Supplemental Table S1. GO Biological Process enrichment analysis was performed to identify biological processes significantly enriched in aged versus young aortas. ECM, calcification, inflammation, and senescence-associated processes were among those significantly enriched. Representative genes showing increased expression with aging were selected from these processes based on their biological relevance for visualization in Figure 1A.

### 2.3. Western Blot Assay

Mice at the indicated ages were euthanized and aortas were harvested, flash-frozen in liquid nitrogen, and stored at −80 °C until use. Frozen aortic tissues were pulverized under liquid nitrogen using Takara BioMasher Standard tubes (Takara, 9791A, Shiga, Japan). Tissue powder was lysed in RIPA buffer (Cell Signaling Technology, 9806S, Danvers, MA, USA) supplemented with protease inhibitor cocktail I (Research Products International, P50600-1, Mount Prospect, IL, USA) for total protein extraction. Equal amounts of protein (15 μg per lane) were separated on 4–20% Criterion™ TGX Stain-Free™ protein gels (Bio-Rad, Hercules, CA, USA) and transferred using the Trans-Blot Turbo System (Bio-Rad, Hercules, CA, USA). Membranes were incubated with the indicated primary and secondary antibodies as previously described [12]. Protein signals were detected using standard chemiluminescence methods. Detailed antibody information is provided in Supplemental Table S2.

### 2.4. Histological Analysis and Morphometric Quantification

For histological analysis, paraffin-embedded cross sections (5 µm) of the ascending aorta and aortic arch were deparaffinized, rehydrated, and stained using standard hematoxylin and eosin (H&E), Masson’s trichrome (StatLab, KTMTR2, McKinney, TX, USA) and Picrosirius Red (Abcam, ab150681, Cambridge, UK) for fibrosis, whereas Alizarin Red (ApexBio, Cat#K1186, Houston, TX, USA) and von Kossa (StatLab, Cat#KTVKOPT, McKinney, TX, USA) staining were used to assess calcification according to the manufacturers’ protocols. Morphometric quantification of fibrosis and calcification was performed using ImageJ software (version 1.54i) and expressed as the fraction of positively stained area to the whole vessel section.

### 2.5. Immunofluorescence Staining and Quantification

Paraffin-embedded cross-sections were deparaffinized, rehydrated, and subjected to antigen retrieval in citrate buffer (pH = 9; Dako, S236784-2, Santa Clara, CA, USA) by heating for 10 min in a pressure cooker. Sections were then blocked with blocking solution (Dako, X0909, Santa Clara, CA, USA) for 30 min at room temperature and incubated overnight at 4 °C with the indicated primary antibodies. After washing three times with phosphate-buffered saline (PBS), sections were incubated with appropriate secondary antibodies for 1 h at room temperature, followed by three additional washes with PBS. Sections were mounted with NucBlue™-containing ProLong™ Glass Antifade Mounting Medium (Thermo Fisher Scientific, P36918, Waltham, MA, USA). Images were acquired using a ZEISS LSM 900 confocal microscope (Carl Zeiss Microscopy GmbH, Jena, Germany) under identical acquisition settings. Quantification of immunofluorescence signals was performed using ImageJ by measuring the fluorescence fraction of positively stained areas as indicated in the figure legends. Detailed antibody information is provided in Supplemental Table S2.

### 2.6. Statistical Analysis

Unless otherwise indicated, animal-level quantification data are presented as mean ± SD. For bulk RNA-seq, differential expression analysis between young and aged aortas was performed using pyDESeq2. Statistical significance was determined using the Wald test, and *p* values were adjusted for multiple testing using the Benjamini–Hochberg false-discovery rate (FDR) procedure. Genes with an adjusted *p* value (padj) < 0.05 were considered significantly differentially expressed. For single-nucleus RNA-seq analyses, gene module scores and Runx2 expression levels between groups were compared using the two-sided Wilcoxon rank-sum test, with *p* values adjusted using the Benjamini–Hochberg FDR procedure.

For histological and immunofluorescence analyses, each data point represents one aorta, with quantitative values obtained by averaging measurements from at least three randomly selected fields of view per specimen, as indicated in the figure legends. Pairwise comparisons between groups were performed using a two-sided Welch’s unpaired *t* test. For Western blot analysis, each data point represents one biological replicate (one aorta). Comparisons among the four independent age groups were performed using ordinary one-way ANOVA followed by Tukey’s multiple-comparisons test. All statistical analyses were performed using GraphPad Prism (version 10.6.1). Statistical significance was defined as *p* < 0.05 unless otherwise specified.

## 3. Results

### 3.1. Aging Activates MAPK14-Associated Calcific and Inflammatory Gene Programs in the Aorta

Vascular aging is characterized by progressive calcification and fibro-inflammatory remodeling; however, the signaling pathways linking these processes remain incompletely defined. To identify candidate pathways, we re-analyzed our previously published bulk RNA-seq dataset of aortas from young and aged mice [11]. This analysis demonstrates increased expression of representative genes within ECM, calcification, inflammatory, and senescence-associated gene sets in aged vessels (Figure 1A). Notably, genes associated with p38 MAPK signaling are also upregulated with aging, suggesting activation of stress-responsive pathways linked to vascular remodeling (Figure 1B). To further assess MAPK14-dependent transcriptional programs in VSMCs, we leveraged our previously published single-nucleus RNA sequencing (snRNA-seq) dataset generated from aortas of *Apoe*^−/−^ mice with or without VSMC–specific *Mapk14* deletion [9]. Although generated in a distinct disease context, this dataset captures key transcriptional programs associated with vascular remodeling. Analysis of these data shows that MAPK14 deficiency results in downregulation of proliferation, inflammatory, fibrotic, and ossification gene modules in VSMCs (Figure 1C–F). Together, these findings indicate that vascular aging is associated with activation of calcific and inflammatory gene programs and suggest that MAPK14 contributes to regulation of these pathways in VSMCs. Among these pathways, activation of calcification-related gene programs prompted us to further investigate osteogenic signaling during aging.

### 3.2. RUNX2 Expression Increases with Aging and Is Reduced by VSMC-Specific MAPK14 Deficiency

**Figure 2:**
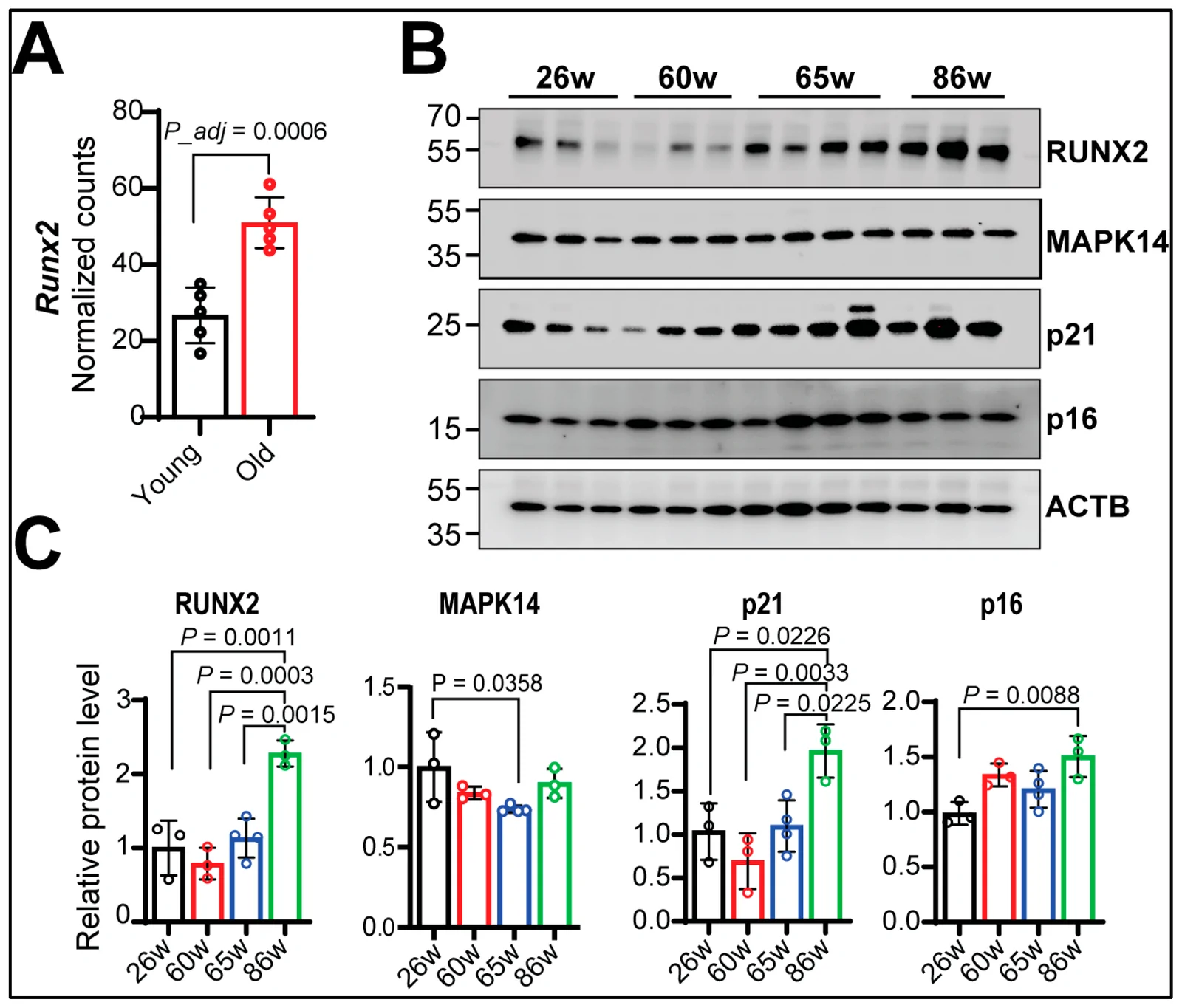
RUNX2 expression increases during vascular aging. (**A**) Bulk RNA-seq analysis shows increased *Runx2* expression in aortas from aged mice compared with young controls (*n* = 5/group). (**B**,**C**) Western blot analysis of RUNX2, MAPK14, and the senescence markers p21 and p16 in aortas from *Apoe*^−/−^ mice at the indicated ages, with the corresponding quantification (*n* ≥ 3/group). Data are presented as mean ± SD. Statistical analyses were performed as described in the Methods Section.

Given that osteogenic signaling is a central feature of vascular calcification, we next examined expression of the master osteogenic regulator Runx2 in the aorta during aging. Consistent with the upregulated ossification gene module revealed by bulk RNA-seq analysis (Figure 1A), Runx2 mRNA expression is increased in aged aortas (Figure 2A). Western blot analysis further demonstrated that RUNX2 protein expression was significantly increased in 86-week-old aortas compared with the 26-, 60-, and 65-week groups (Figure 2B,C). The senescence markers p21 and p16 were also elevated in aged aortas, whereas total MAPK14/p38α protein expression did not show an age-associated increase (Figure 2C).

**Figure 3:**
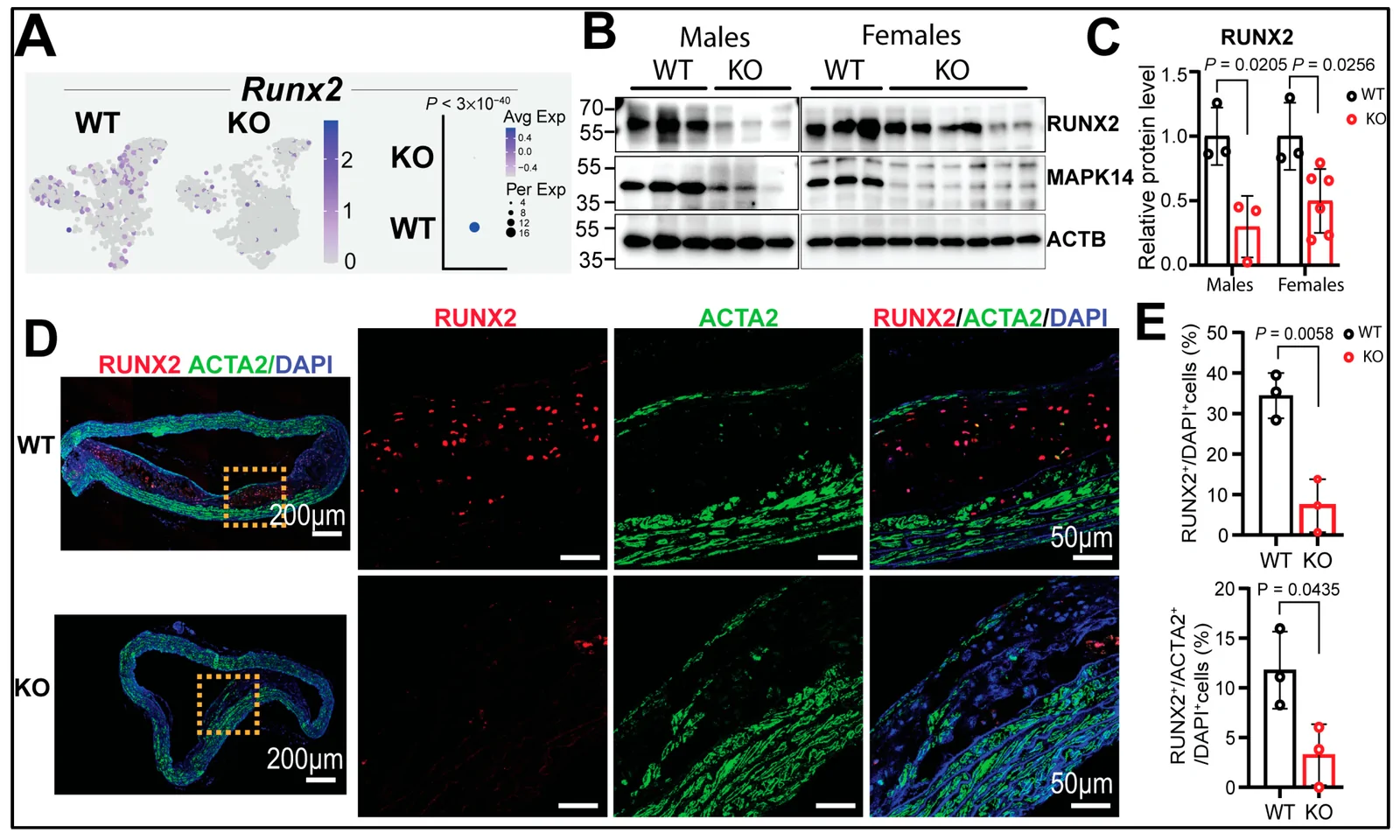
MAPK14 regulates RUNX2 expression in VSMCs during aging. (**A**) snRNA-seq analysis showing reduced *Runx2* expression in VSMCs from KO (SM22-Cre; *Mapk14*^f/f^; *Apoe*^−/−^) compared with WT (SM22-Cre; *Apoe*^−/−^) mice. (**B**,**C**) Reprsentative Western blot analysis and quantification showing reduced RUNX2 protein levels in aortas from KO mice compared with WT controls. Male cohorts consisted of 3 WT and 3 KO mice, whereas female cohorts consisted of 3 WT and 6 KO mice. (**D**,**E**) Representative immunofluorescence staining for RUNX2 and ACTA2 in aged WT and KO aortas, with quantification of total RUNX2-positive cells and RUNX2-positive ACTA2^+^ SMCs, demonstrating significant reductions in both populations in KO aortas compared with WT controls (*n* = 3/group). The dotted rectangle indicates the region shown at higher magnification. Each dot represents one aorta, with values derived from the average of at least three fields of view per sample. Data are presented as mean ± SD. Statistical analyses were performed as described in the Methods Section.

To determine whether MAPK14 regulates RUNX2 expression, we first reanalyzed *Runx2* mRNA expression in VSMCs from our previously published snRNA-seq dataset [9]. *Runx2* mRNA levels are reduced in VSMCs from VSMC-specific *Mapk14* KO mice compared with WT controls (Figure 3A). Consistent with these findings, Western blot analysis of aortas from 26-week-old male and 55-week-old female *Apoe*^−/−^ mice demonstrates significantly reduced RUNX2 protein levels in KO mice compared with their respective WT controls (Figure 3B,C). Immunostaining further demonstrates that the percentages of total RUNX2-positive cells and RUNX2-positive ACTA2^+^ SMCs are significantly reduced in aged KO aortas compared with age- and sex-matched WT aortas (Figure 3D,E). The specificity of RUNX2 staining was confirmed by secondary antibody-only and IgG negative controls (Supplementary Figure S1). Together, these findings support a positive role for VSMC MAPK14 in regulating RUNX2 expression during vascular aging.

### 3.3. Loss of MAPK14 in VSMCs Attenuates Vascular Calcification in Aged Hyperlipidemic Mice

**Figure 4:**
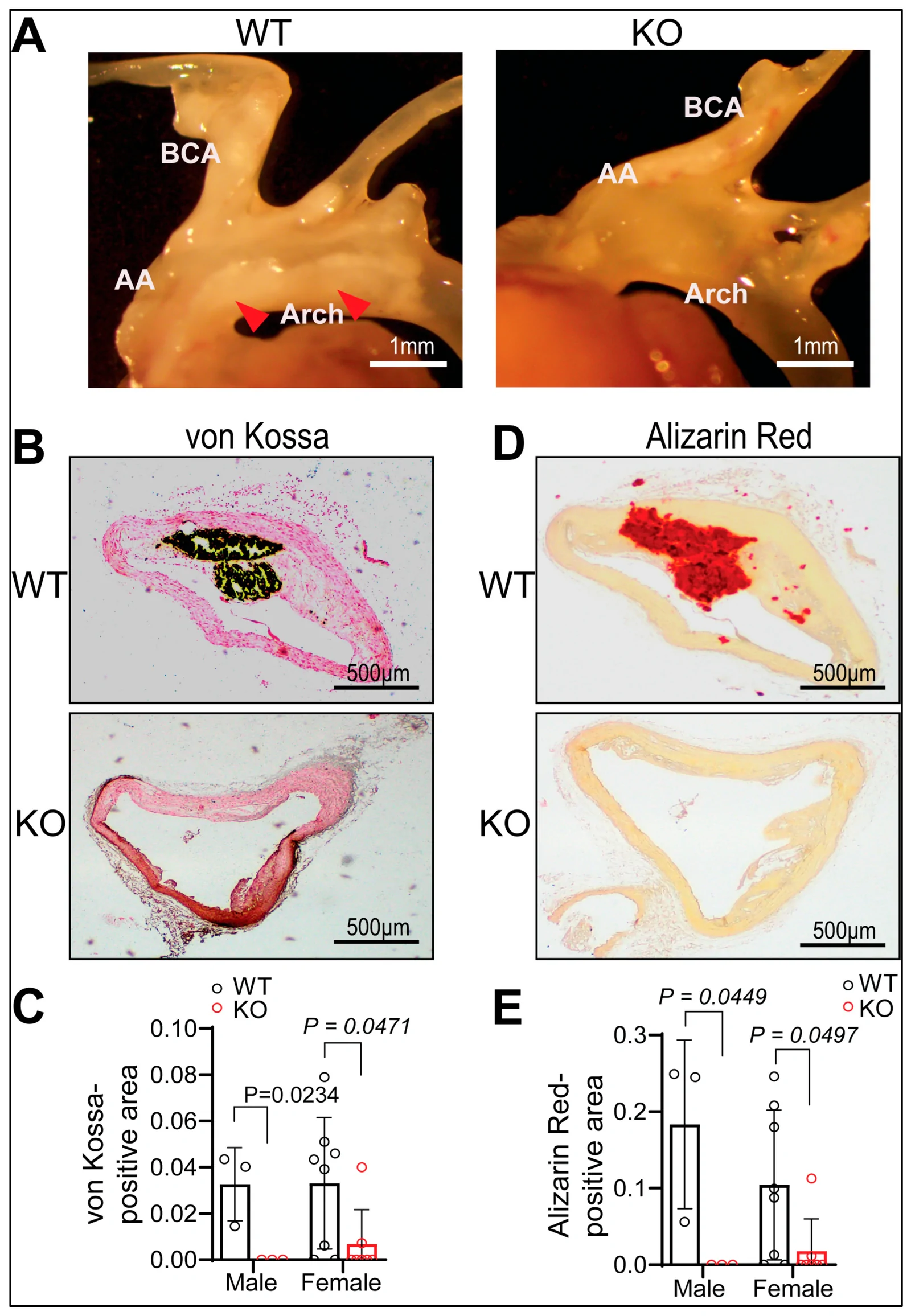
VSMC–specific MAPK14 deficiency attenuates vascular calcification during aging. (**A**) Representative gross images of the aortic arch and ascending aorta from WT (SM22-Cre; *Apoe*^−/−^) and VSMC-specific *Mapk14* KO (SM22-Cre; *Mapk14*^f/f^; *Apoe*^−/−^) mice. AA, ascending aorta; Arch, aortic arch; BCA, brachiocephalic artery. Arrowheads indicate gross vascular lesions. Arrowheads indicate gross vascular lesions. (**B**) Representative von Kossa staining of ascending aortic sections shows reduced calcification in KO mice compared with WT controls; *n* = 3/group for males and *n* = 8 WT and 7 KO for females. (**C**) Quantification of von Kossa-positive area as a fraction of the aortic wall area in male and female mice. (**D**) Representative Alizarin Red staining of ascending aortic sections showing reduced calcium deposition in KO mice compared with WT controls; *n* = 3/group for males and *n* = 8 WT and 7 KO for females. (**E**) Quantification of Alizarin Red-positive area as a fraction of the aortic wall area in male and female mice. Data are presented as mean ± SD. Statistical analyses were performed as described in the Methods Section. *p* values are indicated.

The reduction in RUNX2 expression associated with MAPK14 deficiency in VSMCs prompted us to test whether MAPK14 regulates vascular calcification during aging. To this end, we examined calcification in the aortic arch and ascending aorta of aged (86-week-old) WT and KO mice maintained on a normal chow diet. Age- and sex-matched WT and KO mice were used for all analyses. Gross examination reveals prominent vascular lesions in the aortic arch and ascending aorta of WT mice, which appear reduced in the KO mice (Figure 4A). Histological analysis using von Kossa staining demonstrates decreased mineral deposition in KO mice compared with WT controls, and quantitative analysis confirms reduced calcified area in both male and female mice (Figure 4B,C). Consistent with these findings, Alizarin Red staining further demonstrates reduced calcium deposition in KO mice, which is confirmed by quantitative analysis (Figure 4D,E). Together, these results demonstrate that VSMC-specific MAPK14 deficiency attenuates vascular calcification during hyperlipidemic aging.

### 3.4. Loss of MAPK14 in VSMCs Attenuates Vascular Fibrosis and Inflammation During Aging

**Figure 5:**
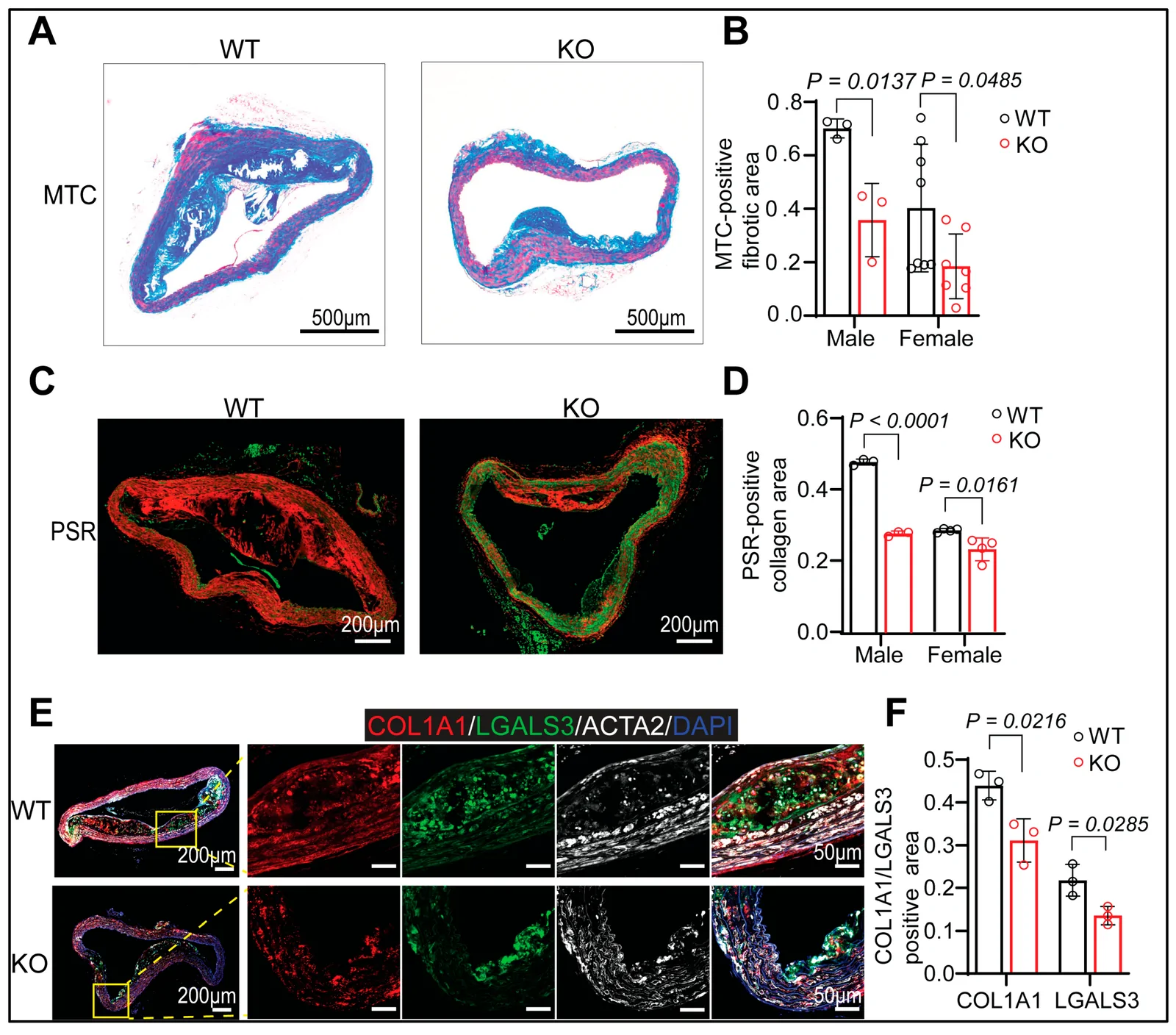
VSMC-specific MAPK14 deficiency attenuates vascular fibro-inflammatory remodeling during aging. (**A**) Representative Masson’s trichrome (MTC) staining of aortic sections from WT (SM22-Cre; *Apoe*^−/−^) and VSMC-specific *Mapk14* KO (SM22-Cre; *Mapk14*^f/f^; *Apoe*^−/−^) mice shows reduced fibrosis in KO mice compared with WT controls; *n* = 3/group for males and *n* = 8 WT versus 7 KO aortas for females. (**B**) Quantification of MTC-positive (blue) fibrotic area as a fraction of the aortic wall area in male and female mice. (**C**) Representative Picrosirius Red (PSR) staining under polarized light shows reduced collagen deposition in KO mice compared with WT controls; *n* = 4/group. (**D**) Quantification of PSR-positive collagen area as a fraction of the aortic wall area in male and female mice. (**E**) Representative immunofluorescence staining for COL1A1 (red), LGALS3 (green), ACTA2 (white), and DAPI (blue) in aortic sections from WT and KO mice demonstrates reduced fibrotic and inflammatory remodeling in KO mice compared with WT controls. Insets show higher-magnification views of the boxed regions; *n* = 3/group. Each dot represents one aorta, with values derived from the average of at least three fields of view per sample. (**F**) Quantification of COL1A1- and LGALS3-positive areas as fractions of the aortic wall area in WT and KO mice. Data are presented as mean ± SD. Statistical analyses were performed as described in the Methods Section. *p* values are indicated.

In addition to calcification, vascular aging is characterized by progressive fibrosis and ECM remodeling. MAPK14 has been linked to fibrosis and inflammation in various vascular disease contexts [8,9]. To determine whether MAPK14 contributes to these processes, we examined fibrotic remodeling in the aortic arch and ascending aorta from aged (86 weeks) WT and KO mice maintained on a normal chow diet. Masson’s trichrome staining shows reduced fibrotic area in KO mice compared with WT controls, which is confirmed by quantitative analysis (Figure 5A,B). Consistent with these findings, Picrosirius Red staining under polarized light demonstrates decreased collagen deposition in KO mice relative to WT mice (Figure 5C,D). To further assess molecular markers of fibrosis and inflammation, we performed immunofluorescence staining for the fibrotic marker COL1A1 and the proinflammatory marker LGALS3. KO aortas show reduced COL1A1-positive and LGALS3-positive areas compared with WT controls, with quantification confirming these reductions (Figure 5E,F). Together, these findings demonstrate that VSMC-specific MAPK14 deficiency attenuates fibro-inflammatory vascular remodeling during aging.

## 4. Discussion

Vascular aging is a major driver of cardiovascular morbidity and is characterized by progressive structural and functional remodeling of the arterial wall. Prior transcriptomic analyses, including our own, demonstrate that aging is associated with activation of inflammatory and stress-related pathways together with ECM remodeling in the aorta. However, the signaling mechanisms linking these aging-associated changes to osteogenic activation and vascular calcification remain incompletely defined. In the present study, we identify VSMC MAPK14 as an important regulator of vascular calcification in the aging aorta. By integrating previously published transcriptomic datasets with genetic and functional analyses, we show that VSMC-specific MAPK14 deficiency reduces RUNX2 expression and attenuates vascular calcification, accompanied by reduced fibrosis and inflammatory remodeling in aged *Apoe*^−/−^ mice. These findings support a model in which VSMC MAPK14 contributes to coordinated osteogenic and fibro-inflammatory remodeling of the aging vascular wall.

VSMCs play a central role in vascular aging through their contribution to vascular calcification. Rather than representing a passive degenerative process, vascular calcification is increasingly recognized as an actively regulated process driven by phenotypic transitions of VSMCs toward osteogenic and matrix-remodeling states [13]. Prior transcriptomic analyses of aged vessels demonstrate activation of inflammatory and stress-associated pathways together with alterations in ECM organization, conditions that favor osteogenic remodeling [11,14]. Consistent with this concept, our analysis demonstrates induction of osteogenic and chondrogenic programs in VSMCs, including increased expression of RUNX2, SOX9, and matrix remodeling genes, consistent with established paradigms of VSMC phenotypic plasticity during vascular calcification [13,15]. Together, these findings support the concept that aging-associated stress signaling promotes osteogenic reprogramming of VSMCs and contributes to vascular calcification during aging.

Vascular calcification is driven by active phenotypic transitions in VSMCs, characterized by acquisition of osteogenic features and expression of bone-related genes [13,16]. In addition, aging promotes activation of inflammatory and stress-associated pathways in the vascular wall, conditions that favor osteogenic signaling and calcific remodeling [14]. Consistent with this, transcriptomic analysis of aged mouse aortas shows increased expression of immune and inflammatory pathways together with altered stress response programs, while largely preserving VSMC lineage identity [11]. Recent studies further demonstrate that sterile inflammatory signaling and senescence-associated pathways act in concert to promote VSMC phenotypic switching and osteogenic activation during vascular aging [17]. Together, these findings support the concept that VSMCs act as key effectors of vascular calcification by integrating stress, inflammatory, and senescence-associated signals into osteogenic remodeling programs. However, the mechanisms by which these pathways are coordinated and reinforced to drive osteogenic gene programs in VSMCs during aging remain incompletely defined.

MAPK14 (p38α) is a stress-responsive kinase involved in the regulation of VSMC phenotypic modulation across multiple vascular disease settings [18,19]. Prior studies, including our own, have shown that VSMC MAPK14 contributes to injury-induced neointimal remodeling, atherosclerotic vascular remodeling, and aneurysm formation, although the downstream transcriptional programs appear context-dependent [7,8,20]. In our previous aneurysm studies, MAPK14 was shown to cooperate with MRTFA-dependent transcriptional signaling to promote VSMC inflammation and senescence, supporting a central role for p38 signaling in maladaptive VSMC activation [9]. In addition, pharmacologic studies in CKD models have linked p38 signaling to arterial calcification, suggesting that this pathway may contribute more broadly to calcific remodeling beyond aneurysm or injury responses [10]. In the present study, we extend these observations to natural vascular aging in hyperlipidemic *Apoe*^−/−^ mice. Reanalysis of aging-associated transcriptomic data shows increased MAPK14-associated signaling in aged aortas, whereas snRNA-seq analysis of VSMC-specific *Mapk14* KO aortas reveals suppression of inflammatory, fibrotic, proliferative, and ossification gene programs. Although our previous study and the present transcriptomic analyses support a role for MAPK14 in inflammatory and fibrotic VSMC phenotypic remodeling, whether MAPK14 drives specific VSMC transitions toward fibroblast-like or macrophage-like states during vascular aging remains to be determined. The reduced fibro-inflammatory remodeling following VSMC-specific MAPK14 deletion may involve both cell-autonomous changes in VSMC phenotype and non-cell-autonomous effects on the vascular microenvironment. These findings support an important role for MAPK14 in coordinating VSMC stress responses with vascular calcification and broader pathological remodeling during aging.

RUNX2 is a master transcription factor required for osteoblast differentiation and bone formation. Aberrant RUNX2 activation in VSMCs has been documented as a key driver of vascular calcification [21]. Increased RUNX2 expression has been observed in multiple vascular diseases associated with pathological calcification, including atherosclerosis, aneurysm, and CKD–associated vasculopathy, where it promotes osteogenic gene programs, VSMC phenotypic switching, and progressive mineral deposition [22,23,24]. Because RUNX2 plays a crucial role in normal bone homeostasis, direct systemic targeting of RUNX2 may carry substantial risk. Therefore, identifying upstream pathways that selectively regulate RUNX2 activation in VSMCs represents an important strategy to limit vascular calcification while preserving normal skeletal homeostasis. Our findings support a functional relationship between MAPK14 signaling and RUNX2 expression in the setting of vascular aging. RUNX2 expression increases in aged *Apoe*^−/−^ aortas, whereas VSMC-specific MAPK14 KO suppresses RUNX2 expression and is accompanied by reduced vascular calcification. However, these findings do not establish direct regulation of RUNX2 by MAPK14, and the precise molecular mechanisms linking MAPK14 signaling to RUNX2 expression remain to be defined. The pronounced reduction in RUNX2 expression in MAPK14 KO mice highlights the therapeutic potential of targeting MAPK14 signaling to suppress pathological osteogenic remodeling. Although selective delivery approaches, including nanoparticle-mediated RNA-based therapies, may provide a feasible strategy to target VSMC MAPK14 signaling, further studies will be required to define the role of MAPK14 in bone homeostasis and evaluate potential systemic effects of MAPK14 inhibition. Collectively, these results suggest that MAPK14-dependent signaling promotes osteogenic activation of VSMCs during aging and may provide a more tractable strategy to limit RUNX2-driven calcific remodeling without directly targeting RUNX2. This mechanism may be particularly relevant in aging and hyperlipidemia, where chronic stress signaling reinforces VSMC osteogenic programs and promotes progressive vascular mineralization.

Several limitations of this study should be acknowledged. Although VSMC-specific MAPK14 deficiency reduces both Runx2 mRNA and RUNX2 protein expression, the precise molecular mechanisms linking p38 signaling to RUNX2 remain to be defined, including whether MAPK14 directly regulates Runx2 transcription or additionally influences RUNX2 protein stability. Because these studies were performed in aged *Apoe*^−/−^ mice, reduced vascular calcification occurs in the context of broader vascular and atherosclerotic remodeling, and the relative contribution of these changes to the calcification phenotype remains to be determined. In addition, the use of SM22-Cre may also target other mesenchymal cell populations, including activated myofibroblasts, which could contribute to the observed phenotype. Despite these limitations, our findings identify MAPK14 as a key regulator of RUNX2-associated osteogenic activation and vascular calcification during aging. These results provide new insight into the signaling pathways linking aging-associated stress responses to vascular calcification and suggest that targeting MAPK14 may represent a therapeutic strategy to limit vascular calcification in aging-associated vascular disease.

## Data Availability

All data supporting the findings of this study are available within the article and its Supplementary Material or from the corresponding author upon reasonable request.

## Supplementary Materials

The following supporting information can be downloaded at: https://www.mdpi.com/article/10.3390/cells15181625/s1, Table S1: Gene list of the selected modules for scRNA-seq; Table S2: Antibody information; Figure S1: Negative controls for RUNX2 immunofluorescence staining; Uncropped Western blot images.
