# Supplementary material for: Smooth Muscle Cell MAPK14 Promotes Vascular Calcification During Hyperlipidemic Aging
Source: Cells. 2026 Sep 8;15(18):1625. doi: 10.3390/cells15181625 (PMC13605429; doi:10.3390/cells15181625)

Supplemental Tables

Table S1. Gene list of the selected modules for scRNA-seq

Proliferation	Inflammation	Fibrosis	Ossification
Igf1	*C1qtnf3*	*Col8a1*	*Cthrc1*
Timp3	*Serpinf1*	*Col3a1*	*Aspn*
Mmp2	*Ctss*	*Col1a1*	*Col1a1*
Adipoq	*Ccn4*	*Col1a2*	*Col1a2*
Tgfb3	*Tnc*	*Lum*	*Igf1*
Cdkn1a	*Il33*	*Col14a1*	*Lox*
Ccn4	*Adipoq*	*Fn1*	*Mmp14*
Thbs1	*Cd44*	*Serpinh1*	*Mmp2*
	Pmp22	*Lox*	*Comp*
	Fcer1g	*Postn*	*Clec11a*
	Fcgr2b	*Fbln2*	*Igfbp5*
	Lpl	*Mfap4*	*Ccn4*
		Col5a1	*Sfrp2*
		Col16a1	*Bmp1*
		Col11a1	*Thbs3*
		Ccdc80	
		Comp	
		Col5a2	
		Loxl2	
		Dpt	
		Ctss	
		Sfrp2	
		Nid1	
		Mmp23	

Table S2. Antibody information

Antibody	Cat number	Working concentration	Supplier
**Primary antibodies for Western blot**			
Anti-ACTB	A5441-100UL	1:2000	Sigma
Anti-p38MAPK	A300-707A-T	1:1000	Bethyl Laboratories
Anti-RUNX2	D130-3	1:1000	MBL Life Science
Anti-p21/WAF1/Cip1	05-345	1:1000	EMD Millipore
Anti-CDKN2A/p16INK4a	ab108349	1:1000	Abcam
**Primary antibodies for immunostaining**			
Anti-ACTA2-Cy3	C6198	1:500	Sigma
Anti-Collagen I	ab270993	1:300	abcam
Anti-Galectin-3 (Mac-2)	14-5301-82	1:200	Thermo Fisher
Anti-Runx2 antibody	Ab192256	1:200	abcam
**Secondary antibodies for Western blot**			
Goat anti-Mouse IgG (H+L) Secondary Antibody, HRP	31430	1:10000	Thermo Fisher Scientific
Goat anti-Rabbit IgG (H+L) Secondary Antibody, HRP	31460	1:10000	Thermo Fisher Scientific
**Secondary antibody for immunostaining**			
Goat anti Rabbit IgG (H+L)	A11008	1:500	Thermo Fisher Scientific

Secondary Antibody, Alexa Fluor 488, Invitrogen			
Goat anti Rabbit IgG (H+L) Secondary Antibody, Alexa Fluor 647, Invitrogen	A21244	1:500	Thermo Fisher Scientific

Supplemental Figures

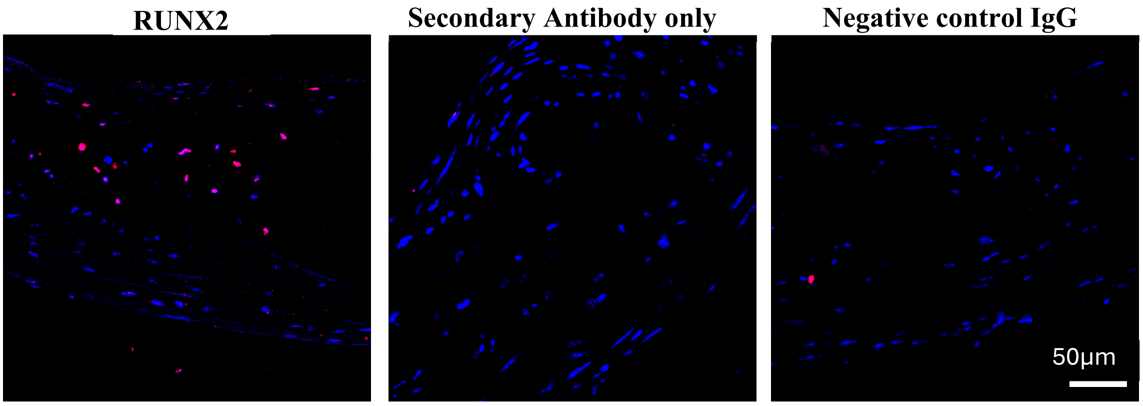

Figure S1. Negative controls for RUNX2 immunofluorescence staining. Representative RUNX2 staining, secondary antibody-only control, and IgG negative control in aged WT aortas. Nuclei were counterstained with DAPI (blue). Scale bar, 50 μm.

Uncropped Western blot images

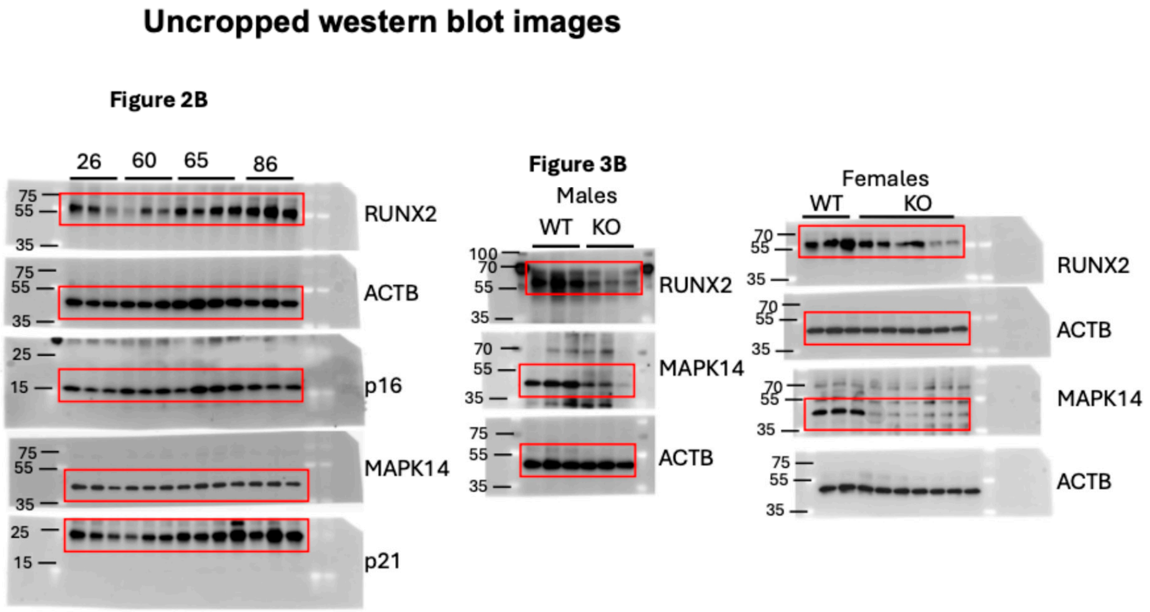

Supplement: Supplementary file 1 [file cells-15-01625-s001.zip › cells-4507671-supplementary.pdf]
